# Type I IFNs enhance human dorsal root ganglion nociceptor excitability and induce TRPV1 sensitization

**DOI:** 10.1172/jci.insight.194987

**Published:** 2025-09-02

**Authors:** Úrzula Franco-Enzástiga, Keerthana Natarajan, Felipe Espinosa, Rafael Granja-Vazquez, Hemanth Mydugolam, Theodore J. Price

**Affiliations:** Center for Advanced Pain Studies, School of Behavioral and Brain Sciences, University of Texas at Dallas, Richardson, Texas, USA.

**Keywords:** Inflammation, Neuroscience, Pain

## Abstract

Type I interferons (IFNs) are critical cytokines for antiviral defense and are linked to painful diseases like rheumatoid arthritis, lupus, and neuropathic pain in humans. IFN-α therapy can cause myalgia, headache, and joint and abdominal pain. Studies in rodent models demonstrate that direct action of IFNs on sensory neurons in the dorsal root ganglion (DRG) promotes hyperexcitability, but rodent behavioral data on IFNs are conflicting, with reports of both pro- and antinociceptive actions. We sought to clarify the action of IFN-α and IFN-β on human DRG (hDRG) nociceptors. We found that IFN receptor subunits IFNAR1 and IFNAR2 are expressed by these neurons, and their engagement induces canonical STAT1 signaling and noncanonical MAPK activation as measured by increased phosphorylation of the cap-binding protein elongation initiation factor 4E by MAPK interacting kinases 1/2 (MNK1/2). Using patch-clamp electrophysiology, Ca^2+^ imaging, and multielectrode arrays, we demonstrated that IFN-α and -β increase the excitability of hDRG neurons with acute and long-term exposure. Type I IFNs prolonged the duration of capsaicin responses, an effect that is blocked by inhibition of MNK1/2 with eFT508, a specific inhibitor of these kinases. This study supports the conclusion that type I IFNs induce hyperexcitability and transient receptor potential vanilloid 1 sensitization when they interact with IFNAR1/2 in hDRG nociceptors.

## Abstract 2

Type I interferons, which are increased in many painful diseases like rheumatoid arthritis, induce increased excitability and TRPV1 sensitization in human pain sensing neurons through MNK1/2 signaling.

## Graphical abstract

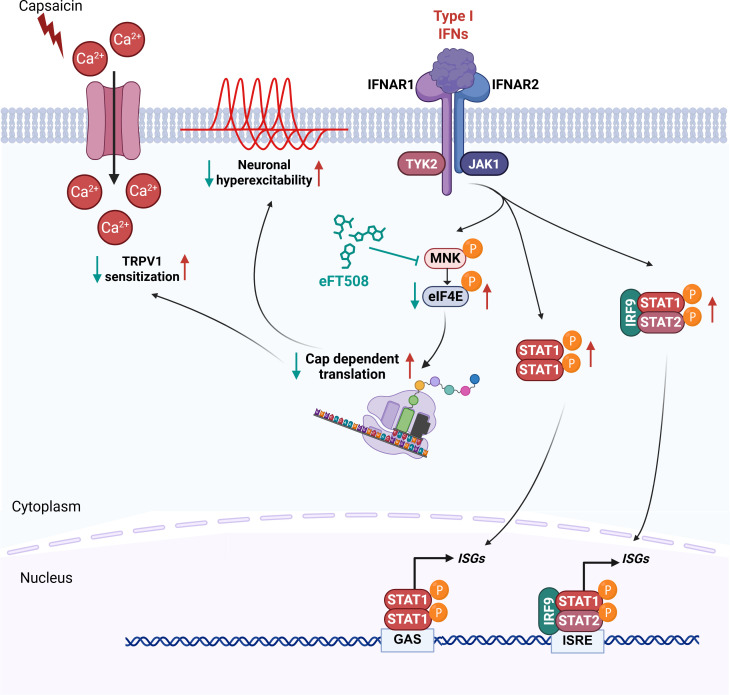

## Introduction

Type I interferons (IFNs) are secreted proteins rapidly induced by viral infection that play a key role in host defense responses (1, 2). They belong to a family of genes contained on chromosome 9 that includes IFN-α, IFN-β, IFN-ε, IFN-κ, and IFN-ω in humans. There are 13 genes coding for human IFN-α (hIFN-α) subtypes that share an amino acid sequence identity ranging from 75% to 99%. Structural differences among type I IFNs account for their varying affinity for the IFN receptor (IFNR) and, consequently, distinct downstream signaling events, such as the phosphorylation of specific signal transducers (3).

Type I IFNs bind to a shared cell surface receptor composed of 2 transmembrane subunits, IFNAR1 and IFNAR2. After the ternary complex assembly, Janus kinase 1 (JAK1) and tyrosine kinase 2 (TYK2), which are associated with the cytoplasmic domains of IFNAR1 and IFNAR2, respectively, activate each other by transphosphorylation and phosphorylate specific tyrosine residues of IFNAR1 and IFNAR2 (4). Phospho-sites then serve as a docking region for effector proteins of the STAT family. Effector proteins propagate downstream signaling (5). The hallmark of IFN signaling comprises STAT1 and STAT2 phosphorylation and the formation of a heterotrimer with IFN regulatory factor 9 (IRF9) to form the interferon-stimulated gene factor 3 (ISGF3). ISGF3 translocates to the nucleus to activate the expression of IFN-stimulated genes (ISGs) (6). ISGs’ functions include inhibition of viral replication by interfering with the viral life cycle, induction of cell death, and activation of innate and adaptive immunity (7). JAKs can induce the formation of STAT1 and STAT3 homodimers that also signal to promote pro-inflammatory responses or limit them, respectively (8). For antiviral responses, primarily phosphorylation of STAT1/2 is required (5). In addition to the JAK/STAT pathway, IFNs can activate noncanonical pathways, such as MAPKs, PI3K/mTOR, and cyclin-dependent kinases (9). Notably, while the role of these pathways in IFN signaling is recognized in certain biological systems, their relevance is highly dependent on specific cell types (5).

A pro-nociceptive effect of IFN-α and IFN-β is supported by experiments in rodents (10–12), by clinical reports on the side effects of type I IFN therapy peginterferon-α2a (13–16), by evidence that ISGs are turned on in the dorsal root ganglia (DRG) of women with neuropathic pain (17), and by increased type I IFN production in painful autoimmune diseases, like rheumatoid arthritis and lupus (18–20). Guided by the hypothesis that IFNs may induce pain during viral infection, our former work in mice showed that type I IFNs induce nociceptor hyperexcitability and pain when applied to the periphery (11). Furthermore, we showed that they engage MAPK interacting kinase (MNK) signaling to the elongation initiation factor 4E (eIF4E) pathway (11), a signaling mechanism that is also linked to nociceptor hyperexcitability in humans with neuropathic pain (21, 22). The MNK-eIF4E cascade participates in the translation regulation of specific mRNAs associated with antiviral response, inflammation, metastasis, synaptic plasticity, and pain (23–25). Recently, we demonstrated that stimulator of interferon genes (STING)-IFN-MNK-eIF4E activation underlies pain sensitization caused by a chemotherapeutic agent in mice (12). In the same line, eIF4E has been shown to increase the translation of the E3 ubiquitin ligase tripartite motif protein 32 (TRIM32), enhancing type I IFN signaling and mechanical hypersensitivity in mice (26). Finally, a recent study demonstrated that persistent type I IFN signaling drives MNK-eIF4E–dependent pain in mouse models of rheumatoid arthritis, with support for similar signaling in clinical samples from patients (20). Accordingly, inhibiting MNK-mediated phosphorylation of eIF4E has proven beneficial in various preclinical chronic pain models, and painful diseases in humans, rendering MNK an important mechanistic target for pharmacological pain relief (11, 20, 21, 24, 25, 27–31).

In contrast with studies suggesting pain induction by IFNs, a recent study showed that CFA-induced inflammation activates STING/IFN-β signaling in nociceptors, leading to decreased nociceptor excitability via upregulation of KChIP1-Kv4.3 (32). Other studies have shown that STING-activated type I IFN signaling reduces neuron excitability by inhibiting the activity of Na^+^ and Ca^2+^ channels (33, 34). In the study of Donnelly and colleagues (33), peripheral administration of IFN-α induced mechanical hypersensitivity that was alleviated by intrathecal administration of IFN-α, suggesting that spinal actions of type I IFNs are antinociceptive, an effect consistent with other studies (35–39). Supporting the notion of type I IFNs as antinociceptive agents, a recent work of Defaye and colleagues demonstrates that IFN-β neutralizing antibody administered intrathecally induces thermal hyperalgesia in mice (32). These seemingly discordant findings led us to test if type I IFNs increase the excitability of hDRG neurons.

The goal of our work was to gain mechanistic insight into the action of type I IFNs on human nociceptors. Here, we demonstrate that type I IFNs induce hyperexcitability and sensitization of human nociceptors as well as STAT activation and increased MNK1/2-mediated eIF4E phosphorylation. Furthermore, we provide evidence that one of the pleiotropic effects underlying IFN-induced nociceptor sensitization involves transient receptor potential vanilloid 1 (TRPV1) sensitization, an effect blocked by the MNK1/2 inhibitor eFT508. We conclude that type I IFNs sensitize human nociceptors, consistent with the observation that DRG IFN signaling is associated with neuropathic pain in women (17) and arthritis pain in animal models and humans (18, 20).

## Results

### IFNAR1 and IFNAR2 mRNAs are expressed in hDRG neurons.

**Figure 1:**
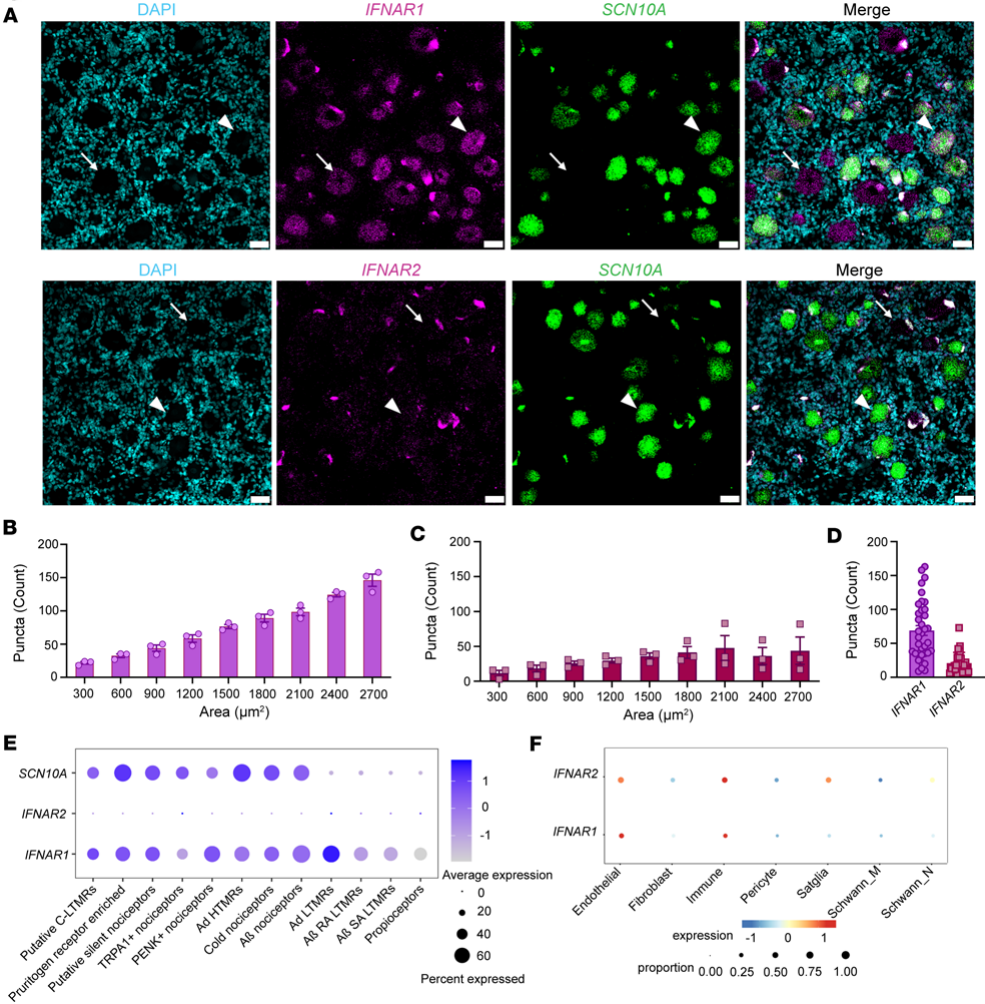
*IFNAR1* and *IFNAR2* mRNAs are expressed in hDRG neurons. (**A**) Representative in situ hybridization images of *IFNAR1* and *IFNAR*2 (magenta) in *SCN10A*(+), neurons (green) in hDRG. Neurons negative and positive for *SCN10A* are indicated by an arrow or arrowhead, respectively. (**B** and **C**) Number of mRNA puncta of IFNAR1 (**B**) or IFNAR2 (**C**) in SCN10A(+) neurons as a function of cell area. (**D**) Number of *IFNAR1* and *IFNAR2* mRNA puncta in *SCN10A*(-) cells across 3 donors in hDRG. In total, 470 neurons were analyzed for IFNAR1 and 314 for IFNAR2. (**E**) Average expression of *IFNAR1/2* in hDRG neurons according to spatial transcriptomic data of hDRG neurons (44). (**F**) Average expression of *IFNAR1/2* in hDRG non-neuronal cell types according to the harmonized atlas of hDRG (43). Scale bar: 50 μm. *SCN10A,* sodium voltage-gated channel alpha subunit 10.

Type I IFNs form a ternary complex with the 2 transmembrane receptor subunits, IFNAR1 and IFNAR2 (40, 41). We assessed the mRNA expression of *IFNAR1* and *IFNAR2* in freshly frozen hDRG from organ donors without a history of chronic pain or neuropathy (Supplemental Table 1; supplemental material available online with this article; https://doi.org/10.1172/jci.insight.194987DS1). In these in situ hybridization experiments, each punctum was considered to represent 1 mRNA of the target. *IFNAR1* was abundantly expressed in all hDRG neurons (Figure 1A). *SCN10A* (+) neurons showed a size-dependent increase in the number of *IFNAR1* mRNAs (Figure 1B), suggesting a similar proportion in every neuron independent of its size, as observed in the mRNA number versus area correlation plots, where the *r* value was similar among all neuronal cell sizes (*r* = 0.62, 0.72, 0.6, respectively; Supplemental Figure 1, A–C). *IFNAR2* was also expressed in all *SCN10A* (+) neurons but at lower levels than *IFNAR1* (Figure 1C). The mRNA number versus area correlation analysis of *IFNAR2* showed a similar trend in small- and medium-sized neurons, and the level decreased in large neurons (*r* = 0.43, 0.21, and 0.03, respectively; Supplemental Figure 1, D–F). Because both subunits are required to induce type I IFN signaling in human cells (42), these results suggest that IFN-α and IFN-β would be expected to have higher activity in small- and medium-sized neurons, likely nociceptors, compared with larger neurons (Supplemental Figure 1, G and H). Both *IFNAR1* and *IFNAR2* were expressed in *SCN10A* (-) neurons, again with a higher proportion of *IFNAR1* compared with *IFNAR2* (Figure 1D). Our results are in line with spatial and single-cell transcriptomics data (43, 44), showing that both *IFNAR1/2* genes are expressed in neurons with higher abundance for *IFNAR1* (Figure 1E) and also consistent with recent data showing a similar pattern for *IFNAR1* and *IFNAR2* mRNA expression in hDRG neurons (20). Both *IFNAR1* and *IFNAR2* were also expressed in non-neuronal cell types, as shown in single-cell sequencing experiments in hDRG (43) (Figure 1F) and in situ hybridization experiments with puncta outside neurons (Figure 1A), aligning to recently published data showing its expression in satellite glial cells (20).

### IFNAR1 and IFNAR2 proteins are present in hDRG neurons from organ donors.

**Figure 2:**
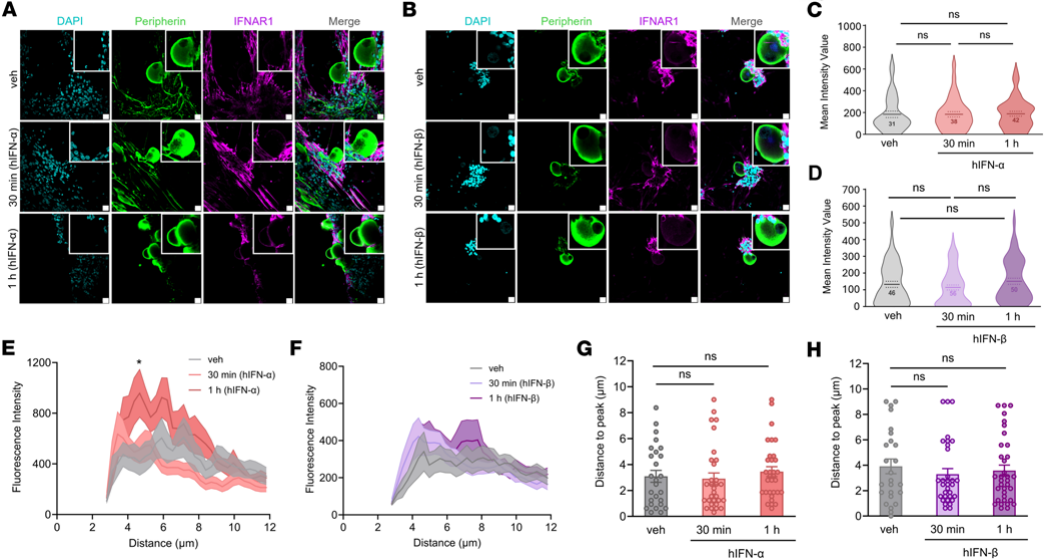
IFNAR1 protein expression in cultured hDRG neurons and response to type I IFN incubation. (**A** and **B**) Representative confocal images of IFNAR1 (magenta) in peripherin-positive neurons (green) 30 minutes or 1 hour after 500 U/mL hIFN-α or hIFN-β treatment, respectively. (**C** and **D**) Neuronal mean gray intensity value of IFNAR1 in dissociated cultures incubated with hIFN-α or hIFN-β, respectively, for 30 minutes or 1 hour. (**E** and **F**) Mean gray intensity values along perpendicular regions of interest (ROIs) hand drawn to assess IFNAR1 cell surface accumulation signal spanning 10 μm from the exterior of the neuron toward the center of the cell upon hIFN-α or hIFN-β stimulation, respectively. (**G** and **H**) Distance to max gray intensity value for IFNAR1 along the perpendicular ROIs hand drawn from neurons stimulated with hIFN-α or hIFN-β, respectively. Data are presented as individual values of mean gray value; treatment mean ± SEM are represented; *N* = 2 organ donors; technical replicates per donor = 2–3 cultures per condition. One-way ANOVA followed by Bonferroni’s test was used to assess group differences in **C**, **D**, **G**, and **H**. *P < 0.05 as determined by 2-way ANOVA followed by Bonferroni’s test in **E** and **F**. Scale bar: 20 μm. veh, vehicle.

**Figure 3:**
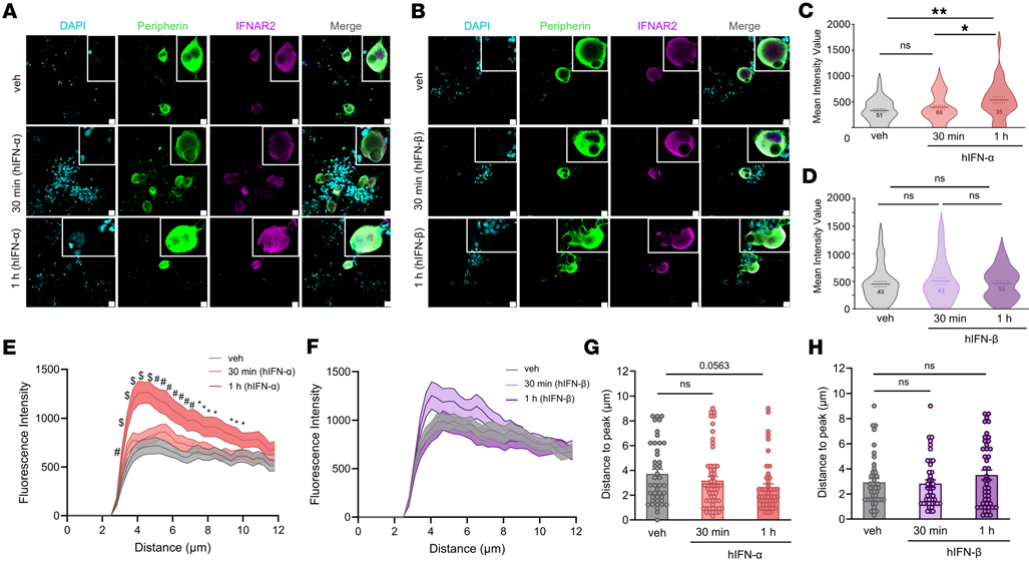
IFNAR2 protein is upregulated by hIFN-α in cultured hDRG neurons. (**A** and **B**) Representative confocal images of IFNAR2 (magenta) in peripherin-positive neurons (green) 30 minutes or 1 hour after 500 U/mL hIFN-α or hIFN-β treatment, respectively. (**C** and **D**) Neuronal mean gray intensity value of IFNAR2 in dissociated cultures incubated with hIFN-α or hIFN-β, respectively, for 30 minutes or 1 hour. (**E** and **F**) Mean gray intensity values along perpendicular ROIs hand drawn to assess IFNAR2 cell surface accumulation signal spanning 10 μm from the exterior of the neuron toward the center of the cell upon hIFN-α or hIFN-β stimulation, respectively. (**G** and **H**) Distance to max gray intensity for IFNAR2 along the perpendicular ROIs hand drawn from neurons stimulated with hIFN-α or hIFN-β, respectively. Data are presented as individual values of mean gray value; treatment mean ± SEM are represented; *N* = 2 organ donors; technical replicates per donor = 2–3 cultures per condition. *P < 0.05, **P < 0.01 as determined by 1-way ANOVA followed by Bonferroni’s test in **C**, **D**, **G**, and **H**. *P < 0.05, ^#^*P* < 0.01, ^$^*P* < 0.001 vs. veh as determined by 2-way ANOVA followed by Bonferroni’s test in **E** and **F**. Scale bar: 20 μm.

Based on our RNAscope findings, we tested for the presence of IFNAR subunits in hDRG neurons on cultures prepared from organ donors treated with vehicle or IFNs using immunocytochemistry (ICC). Previously published RNA-sequencing data show that cultured hDRG neurons at days in vitro (DIV) 4 express both genes *IFNAR1* and *IFNAR2*; therefore, receptors for type I IFN signaling are preserved in our cultures (45). We incubated cultures acutely with 500 U/mL of hIFN-α or hIFN-β (~1–2 ng/mL), a concentration based on previous work on mouse DRG cultures and on other cells (11, 46, 47). In control conditions, IFNAR1 was found to be expressed in peripherin-positive hDRG neurons showing signal in the cytoplasm and in proximity to the cell surface (Figure 2, A and B). Neither hIFN-α nor hIFN-β modified IFNAR1 abundance at 30 minutes or 1 hour after stimulation in peripherin-positive hDRG neurons (Figure 2, A–D) nor its accumulation on the cell surface (Figure 2, E–H). Similar to IFNAR1, in control conditions, IFNAR2 (Figure 3, A and B) was detected in hDRG nociceptors, which were also positive with peripherin, in the cytoplasm and proximal to the membrane. IFNAR1 and IFNAR2 availability proximal to the surface suggests cooperativity among IFNAR subunits to form a ternary complex with IFNs (48), rather than mediating signaling via the activation of IFNAR subunits acting independently of each other (42). hIFN-α increased IFNAR2 abundance in hDRG neurons after 1 hour of incubation (Figure 3, A and C), whereas hIFN-β did not (Figure 3, B, D, F, and H). This is consistent with hIFN-α’s stronger affinity for IFNAR2 (49). These findings are in line with the notion that upon IFN-α binding, IFNAR2 can be internalized and recycled back to the cell surface (50), as suggested by increased IFNAR2 accumulation proximal to the membrane in hDRG nociceptors (Figure 3, E and G). Consistent with our in situ hybridization findings, IFNAR1 and IFNAR2 were also detected in non-neuronal cell types in ICC experiments in hDRG cultures (Figure 2, A and B, and Figure 3, A and B). Together, our results verify that both IFNAR1 and IFNAR2 subunits are translated in hDRG neurons, supporting the idea that endogenous type I IFNs can act directly on human nociceptors.

### hIFN-α and hIFN-β induce canonical type I IFN signaling in hDRG neurons.

**Figure 4:**
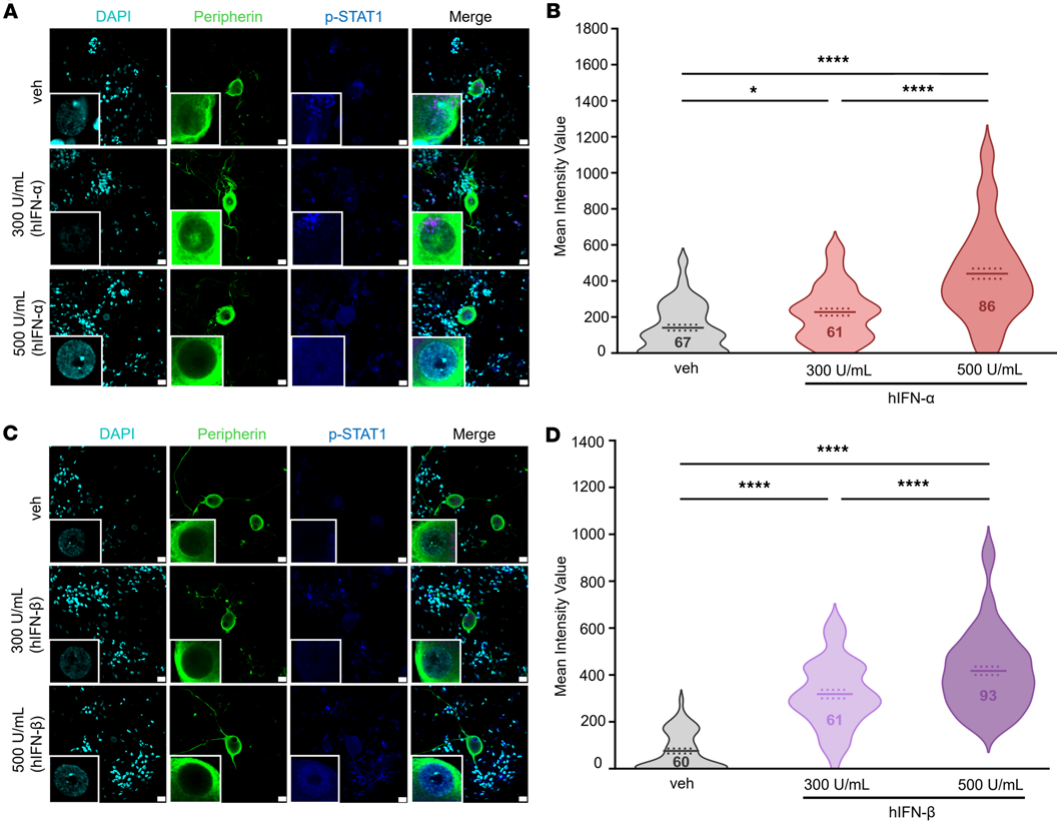
Type I IFN induction of STAT1 signaling in the nuclear compartment of hDRG neurons. (**A**) Representative confocal images of phosphorylated (p-) STAT1 (blue) in peripherin-positive neurons (green) 1 hour after hIFN-α treatment. (**B**) Neuronal nuclear mean gray intensity value of p-STAT1 in dissociated cultures incubated with hIFN-α for 1 hour. (**C**) Representative confocal images of p-STAT1 (blue) in peripherin-positive neurons (green) 1 hour after hIFN-β treatment. An extra channel was used to subtract the lipofuscin autofluorescence signal. (**D**) Neuronal nuclear mean gray intensity value of p-STAT1 in dissociated cultures incubated with hIFN-β for 1 hour. Data are presented as individual values of mean gray value; treatment mean ± SEM are represented; *N* = 2–3 organ donors; technical replicates per donor = 2–3 cultures per condition. **P < 0.05, ****P < 0.0001 as determined by 1-way ANOVA followed by Bonferroni’s test. Scale bar: 20 μm.

The high abundance of type I IFN receptor mRNA and available protein in hDRG nociceptors and our former IFN-mechanistic work in mouse DRG (11) led us to explore the activation of type I IFN signaling induced by hIFN-α and hIFN-β application to hDRG cultures. STAT1 is the primary transcription factor mediating cellular response to type I IFNs and is considered a good indicator of IFNR signaling initiation (51). It forms a complex with STAT2 and IRF9 inducing ISGs. Previously, we showed that type I IFN application to cultured DRG neurons in mice induces JAK1 and STAT1 phosphorylation (11). Here, we used ICC to directly test the nuclear accumulation of STAT1 phosphorylation induced by type I IFNs in hDRG neurons labeled with peripherin. hDRG cultures were incubated with type I IFNs (300 and 500 U/mL) for 1 hour. We found that hIFN-α and hIFN-β induced a concentration-dependent increase in the phosphorylation of STAT1 (Tyr701) in the nuclear compartment of peripherin-positive neurons 1 hour after treatment (Figure 4, A–D), suggesting early canonical downstream signaling induced by direct stimulation of IFNRs. The magnitude of the increase was 2-fold for 500 U/mL hIFN-α and 4.5 times for 500 U/mL hIFN-β.

### Type I IFN drives human nociceptor excitability after acute and prolonged stimulation.

**Figure 5:**
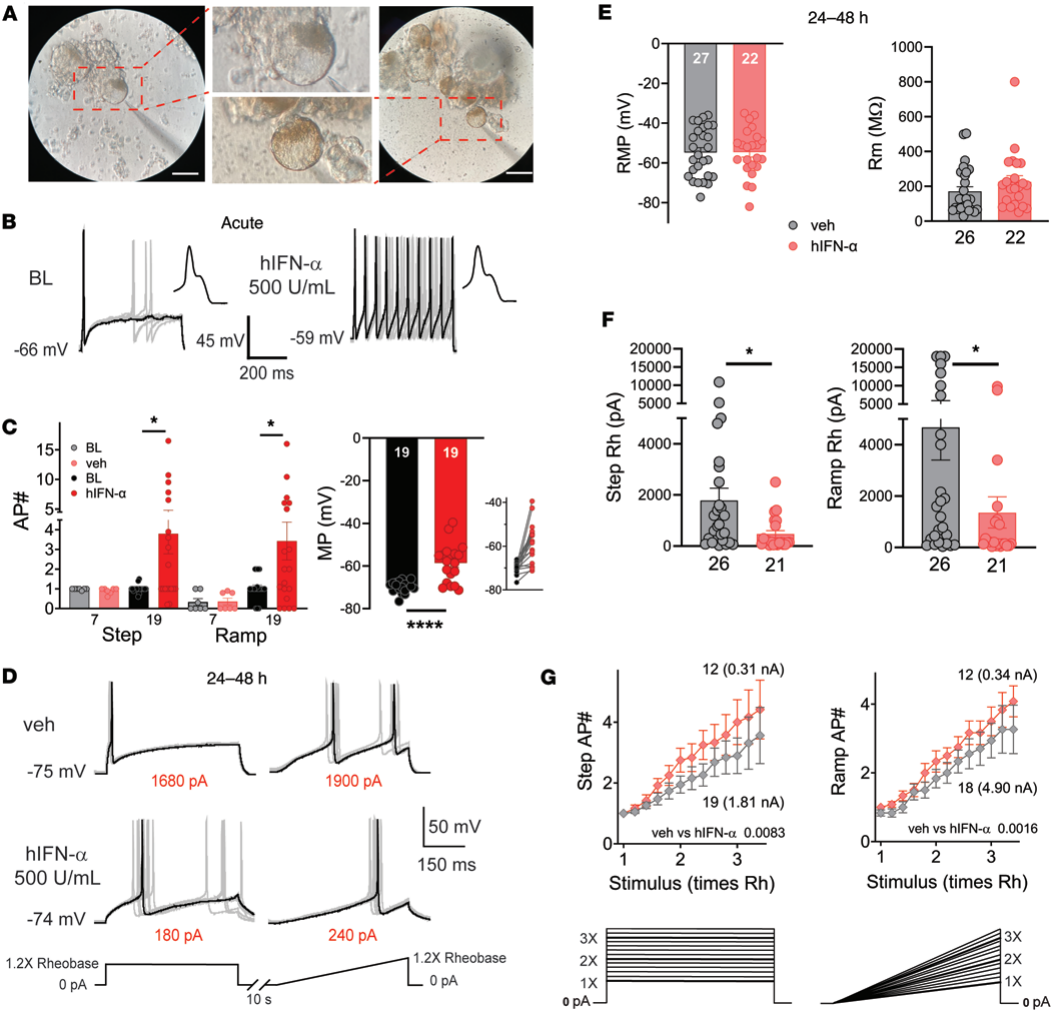
hIFN-α increases the excitability of hDRG neurons. (**A**) Pictures of recorded hDRG neurons. The left picture shows a partially covered hDRG neuron. The right picture shows a neuron devoid of SGCs. The middle pictures are zoomed versions of the left and right pictures. (**B**) AP traces of 10 sweeps before (left) and up to 1 hour after 500 U/mL hIFN-α perfusion (right). In this cell, the number of APs increased almost 10-fold, accompanied by a mild depolarization of 7 mV. Insets show a zoom of the initial APs in a sweep to highlight the presence of the characteristic AP hump in nociceptors. (**C**) AP number (#) in baseline (BL) conditions, and after vehicle (veh) or hIFN-α exposure, using step and ramp protocols (left). Resistance membrane potential (RMP) in BL conditions and after hIFN-α application. The inset highlights that most cells were depolarized in the presence of hIFN-α (right). (**D**) AP traces 24–48 hours after veh (top traces) or hIFN-α incubation (bottom traces). Numbers in red highlight the difference in rheobase (Rh) between the experimental groups. (**E**) RMP and membrane resistance (Rm) in neurons incubated for 24–48 hours with veh or hIFN-α. (**F**) Rh after 24–48 hours of incubation with hIFN-α or veh. (**G**) Number of APs with incremental stimulation intensities after incubation with hIFN-α for 24–48 hours or veh. Numbers in parentheses indicate the average Rh for the corresponding group. Data are presented as mean ± SEM. Paired *t* test was used to assess group differences in **C**, and unpaired *t* test was used in **E** and **F**, and 2-way mixed ANOVA was used in **G**. *P < 0.05, ****P < 0.0001. Scale bar: 50 μm; zoom: 2.3×.

To assess whether the short-term effects of type I IFNs contribute to nociceptor excitability, whole-cell patch-clamp electrophysiological recordings were conducted on cultured hDRG. hDRG nociceptors showed the presence of the hump or shoulder in their action potential (AP) waveform characteristic of nociceptors (52, 53). hDRG neuronal cultures were acutely exposed to 500 U/mL of hIFN-α, a concentration based on p-STAT1 signaling induction observed in our results. Guided by our RNAscope and ICC experiments showing the expression of type I IFNR subunits in small- and medium-sized neurons, electrophysiological recordings were performed on hDRG neurons with these features. Neurons intended for electrophysiology experiments were partially and fully devoid of satellite glial cells (SGCs) (Figure 5A), which initially covered the neurons upon dissociation but detached from them within 1 to 5 days after plating.

To assess the acute effects of type I IFNs,10 baseline APs elicited at 1.2× rheobase (Rh) were recorded. This was followed by perfusion of hIFN-α. In 9/19 (47.4%) of recorded hDRG neurons, hIFN-α increased the number of APs during the first hour (Figure 5, B and C) for both step and ramp stimuli. In these neurons, the AP number increased 2- to 16-fold. In contrast, 0/7 neurons acutely exposed to vehicle showed an increase in AP number. These acute effects are in line with the rapid induction of downstream signaling pathways induced by type I IFNs and with our observations in mouse DRG nociceptors (11). hIFN-α also induced membrane potential depolarization in most cells, with a range of a few millivolts to up to 30 mV (Figure 5C, inset). While some cells that did not respond to hIFN-α with increased AP firing were depolarized, the depolarization was stronger in cells that did respond to hIFN-α. A significant Pearson’s correlation coefficient of 0.623 (*P* = 0.0116) was found when comparing the AP number as a function of the change in membrane potential.

Type I IFNs are known to peak within the first 2 days of viral infection, and then there is a decline facilitating the transition to an adaptive immune response characterized by antibody titers (54). However, in some painful diseases, like lupus, there is a persistent increase in type I IFN signaling that can last for years (19), as well as in DRG from patients with painful rheumatoid arthritis and in the joints of animal models and humans with rheumatoid arthritis (20). We preincubated hDRG cultures with 500 U/mL hIFN-α for 24–48 hours and performed electrophysiological recordings on human nociceptors (Figure 5D). In contrast with acute experiments, this longer hIFN-α treatment did not affect the membrane potential (Figure 5E). However, compared with vehicle treatment, we observed that hIFN-α decreased the step and ramp Rh (Figure 5, F and G). Moreover, when exposed to increasing stimulation intensities (see Methods), neurons incubated with hIFN-α fired a higher number of APs (Figure 5G). Importantly, because the average Rh (numbers in parentheses) was significantly larger in the vehicle group, the difference between groups is more pronounced than they appear in the figure. Most DRG neurons responded to intracellular current injections either by firing 1 or 2 APs or with repetitive firing, consistent with what has recently been shown in a patch-seq study of hDRG neurons (55). In our acute experiments, we classified cells as nonresponsive to IFN treatment if they did not change their spiking pattern from a single spiker. The rheobase of single spiking cells was higher in both step and ramp current than for repetitive spiking neurons, which are presumptive IFN-responsive cells (Supplemental Figure 2A). In long-term treatment experiments we observed that IFN shifted the proportion of cells that were repetitive spikers to a higher proportion than without IFN treatment, which also led to an apparent decrease in the rheobase of single spiking neurons (Supplemental Figure 2B). We did not conduct formal statistical testing on the experiments shown in Supplemental Figure 2B because of the small sample sizes, but given the recent discovery that repetitively spiking cells have properties of C-fiber nociceptors in hDRG, we think that this gives important insight into the likely physiology of these cells given recent patch-seq findings (55). Together these results demonstrate that type I IFNs promote hyperexcitability over short (up to 1 hour) and long (24–48 hours) times in most hDRG neurons.

### The MNK/eIF4E pathway is activated by type I IFNs in hDRG neurons.

**Figure 6:**
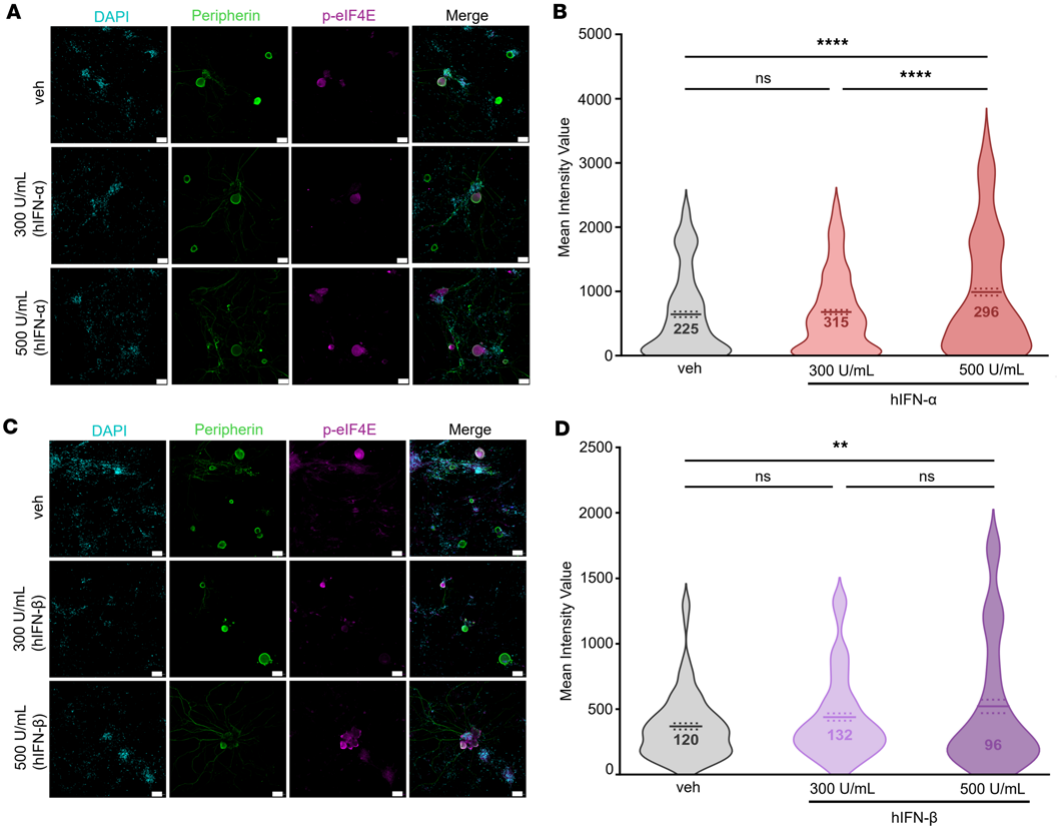
Type I IFNs induce the phosphorylation of eIF4E in hDRG neurons. (**A**) Representative images of p-eIF4E (magenta) in peripherin-positive neurons (green) 1 hour after hIFN-α treatment. (**B**) Neuronal mean gray intensity value of p-eIF4E in dissociated cultures incubated with hIFN-α for 1 hour. (**C**) Representative confocal images of p-eIF4E (magenta) in peripherin-positive neurons (green) 1 hour after hIFN-β treatment. (**D**) Neuronal mean gray intensity value of p-eIF4E in dissociated cultures incubated with hIFN-β for 1 hour. Data are presented as individual values of mean gray value; treatment mean ± SEM are represented; *N* = 2–4 organ donors; technical replicates per donor = 2–3 cultures per condition. **P < 0.01, ****P < 0.0001 as determined by 1-way ANOVA followed by Bonferroni’s test. Scale bar: 50 μm.

Previous work demonstrated that the MNK/eIF4E axis is activated by type I IFNs in mouse DRG neurons and that mechanical nociceptive hypersensitivity caused by type I IFNs in mice is blocked by MNK1/2 knockout or MNK1/2 pharmacological inhibition (11, 20). Using ICC, we tested whether this pathway was induced in hDRG neurons with 300 and 500 U/mL hIFN-α or hIFN-β after 1 hour of treatment (Figure 6). A dose of 500 U/mL of both IFNs increased the phosphorylation of eIF4E (serine 209), a specific biochemical target of MNK1/2 (56) (Figure 6). Since MNK1/2 activation is associated with increased excitability in human nociceptors (21), this finding suggests that this signaling pathway may underlie effects observed in acute and potentially long-term electrophysiology experiments. To further examine these long-term effects, in our subsequent experiments, we tested the involvement of the MNK/eIF4E pathway after a long-term type I IFN incubation.

### MNK/eIF4E pathway contributes to sensitization induced by type I IFNs in hDRG neurons.

**Figure 7:**
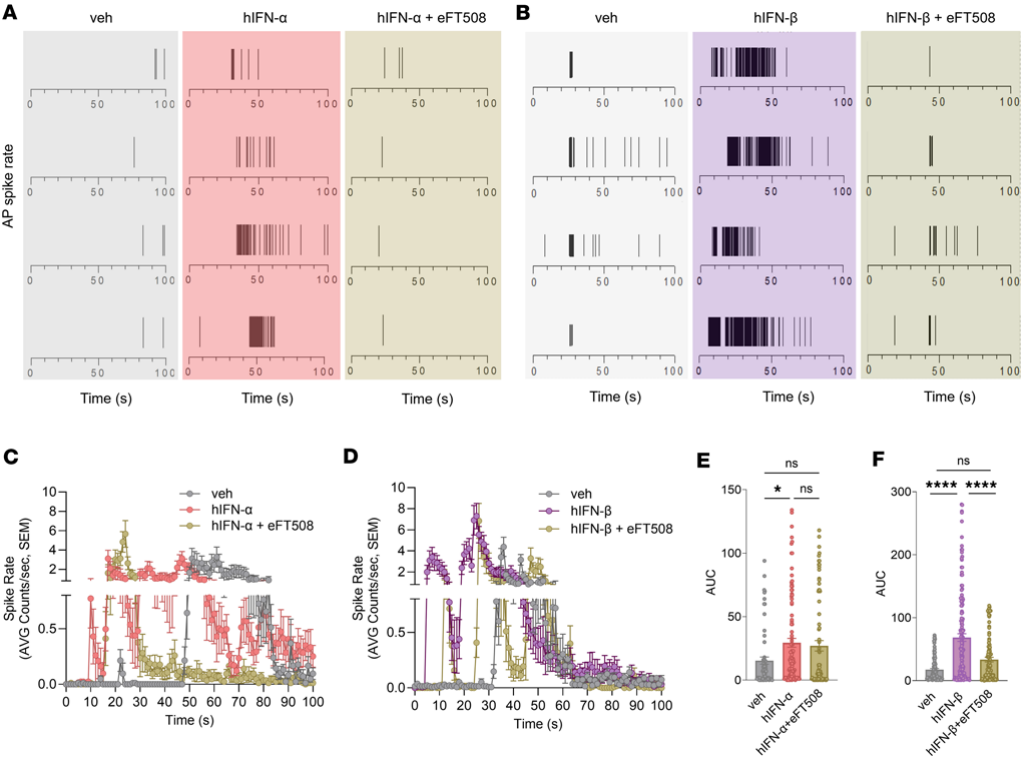
Type I IFNs increase the frequency of APs in hDRG neurons, and the MNK inhibitor abrogates this effect. (**A** and **B**) Representative raster plots showing the frequency (spike rate) of APs to capsaicin (100 nM) in neurons pretreated with vehicle, 500 U/mL hIFN-α or -β, or hIFN-α or -β and 20 nM eFT508 for 24 hours. (**C**) AP spike rate in response to capsaicin of neurons treated with 500 U/mL hIFN-α or hIFN-α and 20 nM eFT508 for 24 hours. (**D**) AP spike rate in response to capsaicin of neurons treated with 500 U/mL hIFN-β or hIFN-β and 20 nM eFT508 for 24 hours. (**E**) AUC as a measure of magnitude of the response in neurons treated with 500 U/mL hIFN-α or hIFN-α and 20 nM eFT508 for 24 hours. (**F**) AUC as a measure of magnitude of the response in neurons treated with 500 U/mL hIFN-β or hIFN-β and 20 nM eFT508 for 24 hours. Data are presented as mean ± SEM. *P < 0.05, ****P < 0.0001 as determined by 1-way ANOVA in **E** and **F**.

To investigate the implications of the MNK/eIF4E pathway in the IFN-induced effects underlying nociceptor hyperexcitability, we utilized a multiwell microelectrode array (MEA) platform to complement our patch-clamp electrophysiological data. MEA technology has the advantage of allowing the functional monitoring of large neuronal populations and has been successfully utilized to characterize human induced pluripotent stem cell–derived nociceptors (57) but has not previously been used with hDRG neurons. APs were recorded in the presence of type I IFNs or vehicle treatment. Independent wells were used to test type I IFNs’ effects in combination with the specific MNK inhibitor eFT508. APs induced by capsaicin were captured after a 24-hour hIFN preincubation period with or without eFT508 over a time course of 100 seconds after 100 nM capsaicin exposure. Examining TRPV1-mediated responses is relevant because recent studies demonstrate a functional link between type I IFNs and TRPV1 (32, 58, 59). Representative raster plots show increased responsiveness to capsaicin in neurons stimulated with type I IFNs, which was dampened in the presence of eFT508 (Figure 7, A and B). Further, an increased AP spike rate in response to capsaicin application was observed with both hIFN-α and hIFN-β, and this effect was abrogated by preincubation with eFT508 (Figure 7, C and D). The magnitude of the AP spike rate response measured as the area under the curve (AUC) showed a statistically significant increase in the cultures stimulated with hIFNs, an effect that was drastically reduced by the application of eFT508 (Figure 7, E and F).

**Figure 8:**
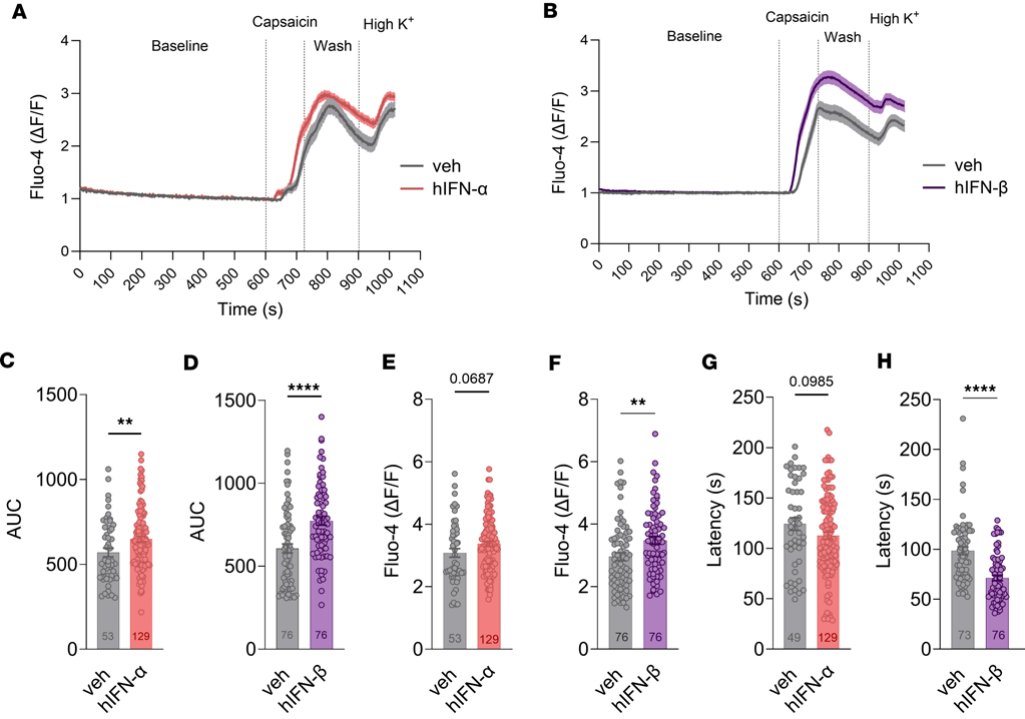
Type I IFN treatment induces sensitization to capsaicin treatment in hDRG nociceptors. (**A** and **B**) Average response traces of hIFN-α and hIFN-β, respectively, or vehicle-treated neurons to capsaicin (200 nM) and high K^+^. (**C** and **D**) Response magnitude to capsaicin of neurons treated with hIFN-α or hIFN-β, respectively, or vehicle. (**E** and **F**) Peak responses to capsaicin in neurons treated with hIFN-α and hIFN-β, respectively, or vehicle. (**G** and **H**) Latency to reach 50% response above baseline to capsaicin in neurons treated with hIFN-α or hIFN-β, respectively, or vehicle. Data are presented as mean ± SEM. **P < 0.01, ****P < 0.0001 as determined by unpaired *t* test in **C**–**H**.

To complement the MEA recordings, Ca^2+^ imaging was used to test nociceptor sensitization via TRPV1 induced by type I IFNs. Preincubation with hIFN-α or hIFN-β for 2.5 hours was sufficient to induce an increased capsaicin (200 nM) response in hDRG neurons (Figure 8, A and B). This statement is based on 3 observations: 1) the magnitude of the response to capsaicin was significantly increased by both hIFN-α and hIFN-β compared with their respective vehicles as measured by the AUC (Figure 8, C and D), 2) the peak Ca^2+^ influx to the cells in response to capsaicin was not statistically changed in the presence of hIFN-α (Figure 8E) but was significantly greater with hIFN-β (Figure 8F), and 3) type I IFNs induced a decrease in the latency of the 50% above baseline fluorescence intensity response to capsaicin with hIFN-β treatment but not hIFN-α (Figure 8, G and H). All the cells used in the analysis were small- to medium-sized neurons, ranging from approximately 100 μm^2^ to 800 μm^2^ (Supplemental Figure 3). These results agree with MEA findings, showing that TRPV1 activity is modulated by type I IFNs in hDRG neurons.

Together, our findings suggest that type I IFNs enhance hDRG nociceptor excitability through a direct activation of IFNAR1/2 engaging the MNK/eIF4E pathway and inducing TRPV1 sensitization.

## Discussion

The experiments described above provide clear evidence for a sensitizing effect of type I IFNs on hDRG nociceptors. First, we provide evidence for the presence of IFNR subunits on the hDRG cell surface and activation of canonical and noncanonical signaling pathways in hDRG neurons upon activation of these receptors. Second, by performing electrophysiological recordings, we found that acute and prolonged IFN exposure induced hyperexcitability of hDRG neurons, as evidenced by an increase in the number of APs fired in response to step or ramp current depolarization and a decreased Rh. Third, the IFN-induced sensitized state was also revealed via Ca^2+^ imaging experiments, which showed that type I IFNs induce TRPV1 sensitization. Fourth, in MEA experiments, an increased frequency of APs in response to capsaicin was triggered by type I IFNs, which was abrogated by the specific MNK inhibitor eFT508. Our findings show that type I IFNs sensitize human nociceptors.

Our initial approach was to determine the expression of IFNAR1 and IFNAR2 in hDRG. IFNAR1/2 participate in the recognition of viral infection like varicella zoster virus, herpesvirus 1 (HSV-1), HSV-2 (60), and SARS-CoV-2 (61). They also participate in painful inflammatory diseases in humans, like rheumatoid arthritis, and neuropathic pain (12, 18, 20, 62). In hDRG neurons, a higher expression of *IFNAR1* mRNA as compared with *IFNAR2* was observed. Imbalance in IFNAR subunit proportion has been observed in different human cell lines, and there is published evidence that the higher abundance of IFNAR1 compensates for the low intrinsic binding of IFN-α to that receptor subunit (50). At the level of protein, both IFNAR1 and IFNAR2 were found in proximity to the cell surface, suggesting that upon IFN stimulation, the ternary complex is formed (48) to facilitate the transphosphorylation of JAK1 and TYK2 with the rapid induction and translocation of p-STAT1 to the nucleus, an effect that we also observed in hDRG neurons. p-STAT1, together with p-STAT2, are a primary requisite for the antiviral response of IFNs (5, 51), suggesting that this canonical pathway is activated directly in nociceptors (11).

With respect to signaling causing sensitization, we observed that eFT508, a highly potent and specific inhibitor of MNK1 and MNK2 (63), abrogated IFN sensitization to capsaicin in hDRG neurons. Our former work in mice showed that exogenous application of type I IFN engaged the MNK/eIF4E pathway via MAPK/ERK phosphorylation, inducing tactile allodynia in mice (11). Furthermore, STING activation triggered endogenous type I IFN release in DRG and sciatic nerves and induced hypersensitivity in mice, an effect that was reduced in MNK1-KO animals (12). Translation regulation via the MNK/eIF4E axis participates in the induction and maintenance of chronic pain, as shown in different preclinical models (11, 25, 27, 64). The phosphorylation of eIF4E controls the translation of mRNAs involved in antiviral response, neuronal plasticity, metastasis, and inflammation (23), actions that have been shown to be therapeutically useful (65). MNK signaling blockade with eFT508 reduces spontaneous activity in nociceptors from humans with neuropathic pain having thoracic vertebrectomy surgery (21). Specifically, eFT508 was shown to reduce AP amplitude and overshoot. Since Nav1.8 is responsible for most of the AP current beyond threshold and AP overshoot in human nociceptors, a reduction of Nav1.8 channel has been hypothesized as a potential consequence of MNK inhibition. Another potential downstream action of MNK inhibition in neurons is a reduction of AP afterhyperpolarization with a potential contribution of hyperpolarization-activated cyclic nucleotide-modulated (HCN) ion channels (21). Interestingly, the MNK/eIF4E pathway is among the axes known to be hijacked by viruses such as HSV-1 (66), suggesting an important role for this pathway in infection. In the same vein, TRIM32, an E3 ubiquitin ligase involved in responses to viral infection, IFN signaling, and pain, is upregulated via activation of the MNK/eIF4E pathway (26). Su and colleagues’ recent work demonstrated that the underlying cause of joint pain is type I IFN signaling–induced hyperexcitability in Gfra3^+^ DRG sensory neurons in mice, with a decisive contribution of IFN-activated MNK/eIF4E pathway (11, 12), which aligns well with our findings on hDRG neurons.

We observed that hIFN-β had a more efficacious effect on p-STAT1 induction as compared with hIFN-α, which can be attributed to IFN-β’s higher affinity for IFNAR1 and IFNRA2 (5). The heightened effects for hIFN-β compared with hIFN-α were also observed in Ca^2+^ imaging and MEA experiments in this work. It is important to highlight that amino acid sequence identity among hIFN-α and hIFN-β is 30%, a difference that likely underlies their specific functional properties and dissymmetrical affinity for IFNAR1 and IFNAR2 (67). The hIFN with the highest affinity binding is hIFN-β (5), showing a superior affinity for IFNAR1 (*K_D_* ~100 nM) and IFNAR2 (*K_D_* ~0.1 nM) (5), compared with the specific hIFN-α that we used in our experiments, hIFN-α2a (*K_D_* ~3.8 μM) (68) (*K_D_* ~1.7 nM) (49). Another explanation for the heightened hIFN-β effect observed in our study could be that IFN-β complexed with IFNAR subunits has a slower off rate than IFN-α. IFN-β remains complexed with IFNAR1 for 100 seconds and with IFNAR2 for 1,000 seconds, while IFN-α has a much faster off rate of 1 second with IFNAR1 and 100 seconds with IFNAR2 (5). Our results support the idea that despite sharing a common receptor and inducing similar effects, type I IFNs are not functionally redundant with respect to the sensory system (48), as they show a differential level of biological activity in IFN signaling in hDRG nociceptors.

Functionally, we demonstrate that hIFN-α induces hyperexcitability in most hDRG nociceptors. Some neurons did not show a response to type I IFN stimulation, but these were single spiking neurons; it is not clear if this lack of response is a lack of a pharmacological activation of the neurons or a physiological property of the cell that cannot change over the short time course of acute treatment (55). Acute and prolonged hIFN-α stimulation enhanced the number of APs indicating hyperexcitability. Further, acute hIFN-α induced membrane potential depolarization but also depolarization of the AP threshold and a reduction of the amplitude of the AP and of the afterhyperpolarization (Supplemental Table 2). Prolonged stimulation with hIFN-α decreased Rh. Type I IFNs can modulate ion channels (46, 69). These changes could be consistent with alterations in the function or in the expression levels of voltage-gated Na^+^, K^+^, or HCN channels. Future experiments will be done to isolate specific ionic currents modified by type I IFNs in human nociceptors. In line with our results, reports on the side effects of hIFN-α, known as peginterferon-α2a, for hepatitis C treatment have demonstrated that it can cause symptoms including diffuse pain, arthralgia, and backache (13). Headaches, myalgia, and joint and abdominal pain have also been reported (14–16).

Contradictory findings have been published in rhesus macaque and mouse DRG neurons indicating a fast suppression of AP firing and increased Rh induced by universal type I IFN, a hybrid of human IFN-α1 (IFNA1) and IFN-α2a (isoform of IFNA2) (33). This effect was shown to be mediated via sodium and calcium channel activity (33, 34). Interestingly, in this previously published study, APs could not be elicited in hDRG nociceptors, but a hyperpolarization effect was observed. In our study, hIFN-α did not induce immediate elicitation of APs but clearly increased nociceptor excitability with short (up to 1 hour) and prolonged (24–48 hours) stimulation. Universal type I IFN hybrid differs from human IFN-α2a used for our experiments, as the carboxy-terminal belongs to the sequence of IFN-α1, whereas the NH2-terminal belongs to that of IFN-α2a. These sequence differences can contribute to variations in the biological activity of the hybrid IFN versus the wild-type IFN and may cause the discrepancy between these findings. In line with our current findings and our previous work on mouse DRG (11), another recent study showed that IFN-α (300 U/mL) induces hyperexcitability in mouse nociceptors 1 hour after incubation (20). Additionally, neuronal hyperexcitability induced by type I IFNs has been observed in other peripheral ganglia and somatosensory systems. For instance, IFNs directly depolarize the membrane potential of bronchopulmonary afferent neurons from nodose ganglia beyond the AP threshold (70). IFN-β has also been demonstrated to increase the membrane resistance and the rate of AP firing in somatosensory neurons of the rat cortex with Ca^2+^-activated K^+^ currents or T-type calcium currents participating in these effects (46). We conclude that our findings are consistent with abundant evidence that type I IFNs increase the excitability of sensory neurons (11, 20) and provide the first evidence to our knowledge that this is the case in human nociceptors.

The work of Defaye and colleagues also differs from the conclusion that type I IFNs sensitize nociceptors. They showed that the activation of the STING/IFN pathway by CFA decreases nociceptor excitability through the upregulation of KChIP1-Kv4.3 (32). They also demonstrated a reduction in the number of TRPV1-positive cells in DRG, and decreased TRPV1 expression was observed in mice expressing the human STING mutation N154S in TRPV1-positive neurons (32). Our MEA and Ca^2+^ imaging findings contradict those findings and support the conclusion that type I IFNs sensitize human nociceptors. Consistent with our findings, it was recently shown that the direct activation of STING induces Trpv1-dependent sensitization in vivo, and in vitro it triggered Ca^2+^ responses in capsaicin-responsive neurons, which were lost in Trpv1-KO mouse cultures (58). Furthermore, viral DNA induces mechanical hypersensitivity via STING-Trpv1 coupling in mouse nociceptors, likely explaining the rapid induction of nocifensive responses to viral dsDNA in mice (58). TRPV1 signaling plays a critical role in the production of antiviral proteins in skin terminals in mice (59), and it has been suggested that proteins like TRPV2 and TRPC1 serve as a port of entry to viruses like HSV-1 into the cell (71). To avoid viral reinfection and spread to bystander cells, TRPV2 is polyubiquitinated by TRIM21 to be degraded in myeloid cells (72). Our results and previous evidence suggest that there is a critical role for the modulation of TRP channels upon viral infection and IFN release. The crosstalk among TRPV1 and type I IFNs is evident in another example. Patients with multiple sclerosis using IFN-β as a treatment develop a flu-like syndrome as the most frequent side effect, presenting with muscle pain, fever, malaise, and weakness. Patients carrying a SNP causing a greater maximal TRPV1 channel response (rs222747) showed higher scores of flu-like syndrome symptoms, suggesting that TRPV1 plays a role in pain induction driven by IFN-β (73).

There are limitations to the work presented here. We did not evaluate transcriptional changes or unbiased protein phosphorylation dynamics induced by type I IFNs in hDRG neurons. While we observed STAT1 activation at a single time point, we did not assess a time course of this effect or examine transcriptional responses in hDRG neurons in response to type I IFNs. These experiments should be prioritized in future studies. Additionally, we did not assess the effect of subcutaneous FDA-approved hIFNs in humans; therefore, we cannot directly address its potential pro-nociceptive effect. Finally, we did not examine any potential sex differences in the actions of type I IFNs on hDRG neurons, though our studies were done on neurons from both male and female organ donors.

In conclusion, our work shows that type I IFNs increase the excitability of hDRG neurons and engage the MNK/eIF4E pathway to induce TRPV1 sensitization. Our findings provide substantial insight into states like viral infection and pathologies like neuropathic pain (17), rheumatoid arthritis (18, 20), and lupus (19) that are linked to increased IFN production and pain that can often become chronic and disabling. We propose that in these diseases type I IFNs likely act directly on nociceptors to potentially promote pain and that targeting these cells with therapeutics like MNK inhibitors would be an effective mechanism to reduce pain caused by increased type I IFN signaling.

## Methods

### Sex as biological variable.

Tissues from both male and female organ donors were used in this work; however, data were not analyzed separately by sex.

### DRG tissue preparation.

DRGs from human organ donors were obtained through a collaboration with the Southwest Transplant Alliance (STA). Right after dissection, lower thoracic or lumbar DRGs from male and female organ donors were either transported in artificial cerebrospinal fluid to be further processed within our facilities or frozen on dry ice right after dissection and stored in a −80°C freezer as has been described in detail previously (74).

DRG donor demographic information is provided in Supplemental Table 1. Our inclusion criteria for this study considered organ donors who exhibited no signs of chronic pain or neuropathy and who did not have a history of medications for chronic pain. Demographic, medical, and prescription drug histories were provided by the STA, based on information gathered through a questionnaire completed by the donor’s next of kin or from available medical records.

### In situ hybridization.

hDRGs were embedded in OCT (Fisher Scientific, catalog 23-730-571) in cryomolds over dry ice and cut into 20 μm sections that were then placed onto SuperFrost Plus charged slides (Thermo Fisher Scientific, catalog 1255015). To allow for the analysis of a diverse population of neurons, 3 sections separated by at least 100 μm from each other were obtained per donor. Cold 10% formalin (pH 7.4) was used to fix the tissues for 15 minutes. This was followed by sequential dehydration in 50% ethanol for 5 minutes, 70% ethanol for 5 minutes, and 100% ethanol for 10 minutes at room temperature. The sections were dried briefly, and ImmEdge PAP pen (Vector Laboratories catalog H-4000) was used to draw hydrophobic boundaries around the tissue. RNAscope probes, commercially available from Advanced Cell Diagnostics (ACD), were used to visualize *IFNAR1* (ACD, catalog 500891), *IFNAR2* (ACD, catalog 490151), and *SCN10A* (ACD, catalog 406291-C2) mRNAs as described previously. In brief, the probes were hybridized at 40°C for 2 hours, followed by amplification. Channel 1 probes were coupled with Cy3 to visualize *IFNAR1* and *IFNAR2* expression and channel 2 with Cy5 to detect SCN10A. Finally, sections were incubated with 1:5,000 DAPI in 1× PBS for 1 minute followed by a rinse with 1× PBS before being air-dried completely. ProLong Gold Antifade Mountant (Thermo Fisher Scientific catalog P36930) was used to coverslip the sections. Sections were imaged at 20× original magnification using the Olympus FV3000 RS confocal laser scanning microscope.

The difference in Gaussian edge detection method was used to perform analysis on ImageJ Fiji version 2.14.0. ROIs were drawn around each neuron, followed by image duplication of the channel with the target probes. Gaussian blur with sigma values of 1 and 2 was applied to the duplicated images. The built-in Image Calculator tool was used to subtract images that allowed for puncta detection using the default threshold function. The number of puncta within each ROI was analyzed using the Analyze Particle feature. Data were plotted using GraphPad Prism V9. Neurons with 1 punctum for IFNAR1/2 and 3 puncta for SCN10A were considered positive for the respective targets.

### hDRG cultures.

Surgically excised lumbar hDRG were obtained from male and female organ donors at STA approximately 2 hours after cross-clamp. We used tissues from both male and female hDRG, as our previous studies showed no sex differences in type I IFN and the IFN/MNK/eIF4E pathways (11). Furthermore, other groups have reported no IFN signaling differences in DRG neurons, though a difference has been observed on microglia; therefore, sex differences at the functional level were not expected, and we did not test for them. Right after dissection, the hDRGs were transported in bubbled NMDG-aCSF pH 7.4 (93 mM NMDG, 2.5 mM KCl, 1.25 mM NaH_2_PO_4_, 30 mM NaHCO_3_, 20 mM HEPES, 25 mM glucose, 5 mM ascorbic acid, 2 mM thiourea, 3 mM sodium pyruvate, 10 mM Mg_2_SO_4_, 0.5 mM CaCl_2_, 12 mM N-acetylcysteine; osmolarity 310 mOsm) on ice to our facilities (75). The hDRGs were minced into small pieces using sterile scissors, immediately transferred to 5 mL of prewarmed (37°C) STEMxyme 1 (Worthington Biochemical, catalog LS004106) solution containing 1 mg/mL STEMxyme in HBSS, 0.1 mg/mL DNase I (Worthington Biochemical, catalog LS002139), and 10 ng/mL recombinant human β-NGF (R&D Systems, catalog 256-GF) and incubated at 37°C in a gently shaking water bath for 8–9 hours for ICC and Ca^2+^ imaging. The tissue was triturated using fire-polished sterile glass Pasteur pipettes with decreasing tip diameters until the tissue was completely digested. The cell suspension was passed through a 100 μm cell strainer (VWR, catalog 21008-950) and then slowly added to 4 mL of 10% BSA to create a gradient, which was then centrifuged (900*g* for 5 minutes, using a profile of 9 for acceleration and 5 for deceleration) at room temperature (RT). Neurons were resuspended in BrainPhys media (STEMCELL Technologies, catalog 05790) supplemented with 1% penicillin/streptomycin, 1% GlutaMAX (United States Biological, catalog 235242), 2% NeuroCult SM1 (STEMCELL Technologies, catalog 05711), 1% N-2 Supplement (Thermo Fisher Scientific, catalog 17502048), 2% HyClone Fetal Bovine Serum (Thermo Fisher Scientific SH3008803IR), 25 ng/mL recombinant human beta-NGF (R&D Systems, catalog 256-GF), 0.15 mg/mL 5-Fluoro-2′-deoxyuridine (Sigma-Aldrich, catalog F0503- 100MG), and 0.35 mg/mL Uridine (Sigma-Aldrich, catalog U3003-5G). hDRG neurons were plated according to the technique for which they were intended, as specified below. The cell cultures were maintained at 37°C with 5% CO_2_. Culture medium was changed every other day.

For electrophysiological recordings and MEA experiments, hDRGs were dissociated as above but with slight differences. hDRGs were weighed and 15–25 mg of minced hDRG was added per milliliter of dissociation solution and incubated at RT on a nutator shaker rotating at 62 rpm. Cells were dissociated into 2 batches. The first cell batch was obtained following 90–120 minutes of initial incubation. Fresh dissociation solution was added to the remaining tissue for an additional 30–60 minutes. At the expense of a lower yield, these shorter periods of dissociation at RT were used to avoid overdigestion and reduce cell membrane damage. This allowed for long recording periods (up to 2 hours) when necessary. Cells were stored in an Eppendorf tube at RT until they were plated. Cells were plated inside cell culture inserts (IBIDI, catalog 80209) attached to a 12 mm coverslip (2–4 per day). At DIV 2–5, cells were exposed to 500 U/mL hIFN-α during electrophysiological recordings (acute) or preincubated 24–48 hours before recordings.

### ICC and image analysis.

Purified recombinant human hIFN-α2a (catalog IF007) and hIFN-β1a (catalog IF014) were purchased from MilliporeSigma. hDRG neurons were cultured on 8-chamber cell culture slides coated with 0.01 mg/mL poly-d-lysine (MilliporeSigma, catalog P7405-5MG) and allowed to adhere for 2 hours at 37°C and 5% CO_2_. The chambers were then flooded with 0.65 mL of medium. hDRG neuronal dissociated cultures (DIV 5–6) were incubated with 300 or 500 U/mL of IFN or vehicle at 37°C with 5% CO_2_. IFNs were applied to hDRG cultures for 30 minutes and 1 hour for ICC experiments. For the immunodetection of p-eIF4E, p-STAT1, IFNAR1, and IFNAR2 in cultured neurons, hDRG cultures were fixed with 10% formalin (Thermo Fisher Scientific, catalog 23-245684) at pH 7.4 for 10 minutes. The chambers were then rinsed 3 times with 0.1 M phosphate buffer (PB) and blocked with 10% NGS and 0.3% Triton X-100 in 0.1 M PB for 1 hour at RT. Next, they were incubated overnight with the primary antibodies anti–p-STAT1 (Tyr701, 1:500; Cell Signaling Technology, catalog 9167), or anti–p-eIF4E (1:2000, Abcam, catalog ab76265), or anti-IFNAR1 (1:500, ABclonal, catalog A18594), or anti-IFNAR2 (1:200, Abcam, catalog ab56070) and α-peripherin (1:1,000, EnCor Biotechnology, catalog cpca-peri). After rinsing 3 times with 0.1 M PB, the cultures were incubated with secondary antibodies goat anti-chicken IgY (1:2,000, H+L, Alexa Fluor 488, Invitrogen, catalog A11039) and goat anti-rabbit IgG (1:2,000, H+L, Alexa Fluor 647, Thermo Fisher Scientific, catalog A21245). The chambers were rinsed 3 times with 0.1 M PB and mounted with ProLong Gold Antifade Mountant (Thermo Fisher Scientific catalog P36930). Slides were examined and imaged at 20× original magnification for p-eIF4E and 40× for p-STAT1 immunodetection using an Olympus FV3000 RS confocal laser scanning microscope. Negative controls were performed by the omission of the primary antibody, which resulted in the absence of immunofluorescence. Images were analyzed using Olympus CellSens Dimension software version 1.18.16686.0 by drawing ROIs on individual neuronal cell bodies and acquiring mean gray intensity values for the targeted fluorophores. An extra channel (Alexa Fluor 555) was used to subtract the lipofuscin autofluorescence signal. To obtain cell surface IFNAR1 and IFNAR2 fluorescence profiles, a line (ROI) perpendicular to the cell surface was hand-drawn on ImageJ on each cell culture stained for IFNAR1 and IFNAR2 stimulated with type I IFNs or vehicle. This line started outside the cell and was extended approximately 10 μm toward the cell center. Mean gray intensity values along the ROIs were plotted for each protein. To avoid nonspecific signal outside the cells of interest, values below 5% of the maximum mean gray intensity value per cell were considered 0.

### Ca^2+^ imaging and analysis.

hDRG neurons were cultured on 12 mm glass coverslips coated with 0.1 mg/mL poly-d-lysine hydrobromide (Sigma-Aldrich, catalog P7405). After pretreatment with hIFN-α or hIFN-β, cells were washed once with medium and loaded with 10 μM Fluo-4 AM (Thermo Fisher Scientific, catalog F142010) with 0.04% Pluronic F-127 (Thermo Fisher Scientific, catalog P3000MP) in HBSS for 30 minutes prior to imaging. The coverslip was mounted onto an imaging chamber (Warner Instruments, catalog RC-25). Cells were washed with recording solution (130 mM NaCl, 4.2 mM KCl, 1.1 mM CaCl_2_, 1 mM MgSO_4_, 0.45 mM NaH_2_PO_4_.H_2_O, 0.5 mM NaH_2_PO_4_.7H_2_O, 20 mM glucose, and 10 mM HEPES; osmolarity 300–310 mOsm; and pH 7.4 adjusted with *N*-methyl-d-glucamine) for 5 minutes at a flow rate of 1 mL/min followed by 10 minutes of baseline recording. A high-K^+^ solution was made by adjusting KCl to 50 mM and NaCl to 84.2 mM in the recording solution. Cells that did not fluctuate during baseline recording and exhibited 10% change in response to 200 nM capsaicin were considered for analysis. Calcium responses were captured using an Olympus IX83 inverted microscope at magnification of 10× and MetaFluor for Olympus imaging software (version 7.10.5.476) (76). Elve Flow MUX Distribution 12-way Bidirectional Valve was used to perfuse solutions over the cells. All recordings were performed within DIV 1–4 at RT.

### MEA.

Primary human sensory neurons were procured and dissociated as described above. Multiwell microelectrode array culture plates (6-well Cytoview, M384-tMEA-6W, Axion Biosystems) were prepared by coating the center of the array with 7 μL 0.01% poly-l-ornithine (PLO; EMD Millipore Corp, catalog A-004-C). The PLO was left overnight at RT. The following day, PLO was removed, and each well was washed 3 times with sterile deionized water and allowed to dry inside the culture cabinet. Once the wells were completely dry, 7 μL laminin (iMatrix silk-511, Matrixome Inc., catalog 892021, at 1:50 in DPBS without calcium or magnesium) was added to the center of the array to ensure that the cells adhered to the area where the electrodes were located. The MEA was maintained at 37°C under 5% CO_2_ for 2 hours. Immediately prior to seeding cells, laminin was removed, and a 7 μL droplet with approximately 300 neurons was added to the center of the well. Cell attachment was verified using a phase-contrast microscope, and the well was flooded with 1 mL of supplemented culture medium, as described in the *hDRG cultures* section. The wells were left inside an incubator and maintained with a 50% medium change every other day.

At DIV 6, each 6-well MEA was taken to a Maestro Pro acquisition system (Axion Biosystems), and AP waveforms were registered for 15 minutes under controlled environmental conditions (humidity, 5% CO_2_, and 37°C) via the enclosed hardware setup. Data were sampled using AxIS software (Axion Biosystems) from raw voltage traces with a bandpass filter from 200 to 3,000 Hz. APs were detected from the filtered voltage trace, and individual units were sampled with an adaptive 5.5 SD sliding threshold window in AxIS Navigator Version 3.7.2.8. For the experiment, 15 minutes were sampled prior to intervention, after which 50% of the medium (500 μL) was removed per well and mixed into a final concentration for each assigned group as follows: a) 500 U/mL hIFN-α, b) 500 U/mL hIFN-β, c) vehicle for hIFN-α treatment, d) vehicle for hIFN-β treatment, e) hIFNs + eFT508 25 nM,and f) vehicle for hIFNs + vehicle for eFT508. Each group consisted of 4 independent wells derived from duplicate experiments and multiple donors (Supplemental Table 1). At 24 hours of treatment, the MEA was registered inside the Maestro Pro system to assess APs after the addition of 100 nM capsaicin to each well.

Files were converted from.spk to.nex format using the Axion Data Export tool (Axion Biosystems) and analyzed in NeuroExplorer (Plexon, Inc., version 5.425) to assess the 2 components. First, rate histograms of the evoked spikes were created from a per-electrode basis in 50 ms bins and plotted in Excel for each group to extract the latency to the first spike after the addition of capsaicin. Then, 1-second bins were used to plot the temporal effect for each group derived from the addition of capsaicin.

### Electrophysiological recordings.

hDRG neurons were recorded 2–7 days after plating (see dissociation details above). The electrophysiological properties of hDRG neurons have been reported to be stable within this range of DIV (52, 55). Moreover, repetitively spiking, single-spiking, and burst-spiking neurons have been shown to be present within this time range (55). The external solution contained (in mM) 125 NaCl, 3.6 KCl, 2 NaH_2_PO_4_, 2.5 CaCl_2_, 2 MgCl_2_, 25 NaHCO_3_, and 11 dextrose. Whole-cell patch-clamp was performed using borosilicate capillaries pulled with a P-97 flaming-brown micropipette puller (Sutter Instruments). The pipettes had a resistance of 2–4 MΩ, when using an internal solution containing (in mM) 120 K-glutamate, 2 KCl, 8 NaCl, 0.2 EGTA, 14 Na_2_-phosphocreatine, 2 Mg-ATP, 0.3 Na-GTP, and 10 HEPES-K (pH 7.3, 295 mOsm). Recordings were obtained using an Axopatch 200B amplifier (Molecular Devices). Neurons were visualized using a Nikon Eclipse Ti inverted microscope equipped with Nikon Advanced Modulation Contrast. Acquisition was done at 10–20 kHz, and data were filtered at 5 kHz and analyzed offline using Clampfit Analysis Suite 11 (Molecular Devices), GraphPad Prism 11, and Microsoft Excel 2025.

After achieving whole-cell configuration, cells were held near resting potential for at least 5 minutes to allow for dialysis of the pipette internal solution. Afterward, when needed, cells were held at –70 mV by injecting current of the appropriate sign and amplitude. Depending on the experiment, one or several of the following protocols were applied: For rheobase evaluation, first, a gross determination of AP firing was made using 10–200 pA incremental steps (500 ms duration), and then a baseline subthreshold current with incremental steps of one-tenth of the apparent Rh in successive sweeps were used. The Rh was considered the step where the first AP was fired and where APs were consistently fired with repeat step stimulation. The maximum stimulation intensity used to test Rh was 18 nA. This maximum value was considered the Rh for cells that did not fire APs. To further determine the excitability characteristics of each cell, they were subjected to 12 incremental steps of magnitude one-fifth that of a baseline set at 1.2× the Rh. Both step and ramp stimulations separated by 10 seconds in each sweep were applied with intersweep intervals of 20–30 seconds. For acute exposure to hIFN-α, 10–15 baseline sweeps were recorded. Then, 500 U/mL of hIFN-α was added, and cells were constantly recorded for up to 1 hour. Acute experiments were paired and recorded from the exact same cell, before and after hIFN-α. Of note, most of the neurons showed a response to hIFN-α within 20–30 minutes. For the nonresponder cells, we waited 1 hour to capture a response. If no response was recorded after this cutoff, the cell was classified as nonresponder.

### Statistics.

Data were plotted and analyzed using GraphPad Prism version 10.0.2232. All data are shown as mean ± SEM. A 2-tailed *t* test or 1-way or 2-way ANOVA followed by Bonferroni’s test was used to analyze data. The test used is indicated for each experiment in the figure legends. *P* < 0.05 was considered statistically significant.

### Study approval.

Human tissue procurement procedures were approved by the Institutional Review Board at the University of Texas at Dallas. The Southwest Transplant Alliance (STA) obtained informed consent for research tissue donation from first-person consent (driver’s license or legally binding document) or from the donor’s legal next of kin. Policies for donor screening and consent are those established by the United Network for Organ Sharing (UNOS). STA follows the standards and procedures established by the US Centers for Disease Control (CDC) and are inspected biannually by the Department of Health and Human Services (DHHS). The distribution of donor medical information is in compliance with HIPAA regulations to protect donor privacy.

### Data availability.

The data supporting the findings reported in the current manuscript are included in the Supporting Data Values file.

## Author contributions

UFE and TJP conceived the idea and designed the research project and experiments; UFE and KN performed IHC and in situ hybridization; FE performed electrophysiological recordings; RGV performed microelectrode recordings; KN performed Ca^2+^ imaging; UFE, KN, FE, RGV, and HM analyzed data; and UFE and TJP wrote the paper. All authors edited the paper and provided input to the final manuscript.

## Supplementary Material

Supplemental data

Supporting data values

## Version 1 09/02/2025

In-Press Preview

## Version 2 10/08/2025

Electronic publication
