# Supplementary material for: Type I IFNs enhance human dorsal root ganglion nociceptor excitability and induce TRPV1 sensitization
Source: JCI Insight. 2025 Sep 2;10(19):e194987. doi: 10.1172/jci.insight.194987 (PMC12513479; doi:10.1172/jci.insight.194987)
Supplement: Supplemental data [file jciinsight-10-194987-s174.pdf]

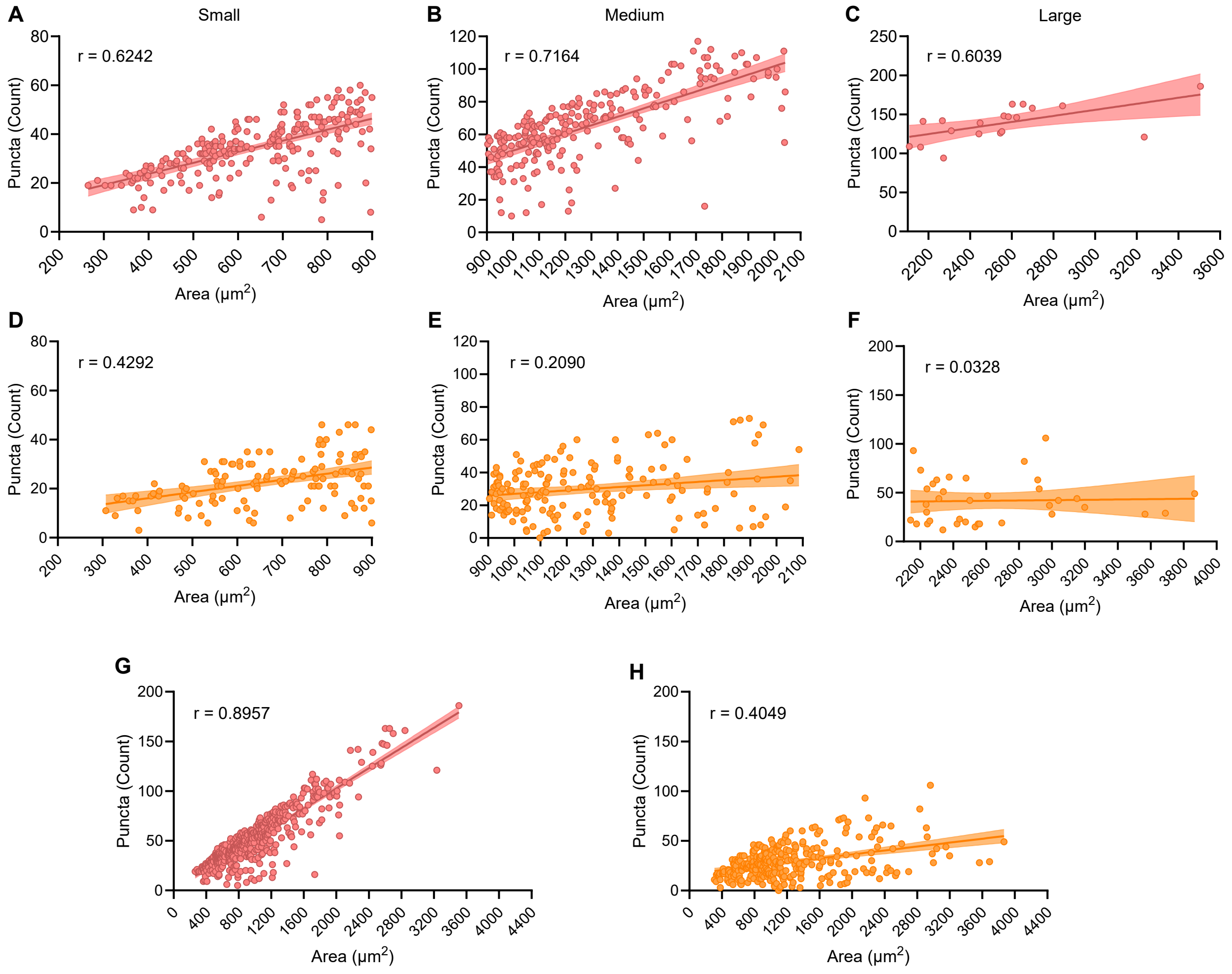

Supplemental Figure 1. Correlation between number of *IFNAR1* and *IFNAR2* mRNA puncta and hDRG neuronal cell area. (**A-C**). Correlation between *IFNAR1* mRNA expression and small (**A**), medium (**B**), and large (**C**) cell sizes. (**D-F**) Correlation between *IFNAR2* mRNA expression and small (**D**), medium (**E**), and large (**F**) cell sizes. (**G**) Correlation between *IFNAR1* mRNA expression and all neuronal cell sizes. (**H**) Correlation between *IFNAR2* mRNA expression and all neuronal cell sizes.

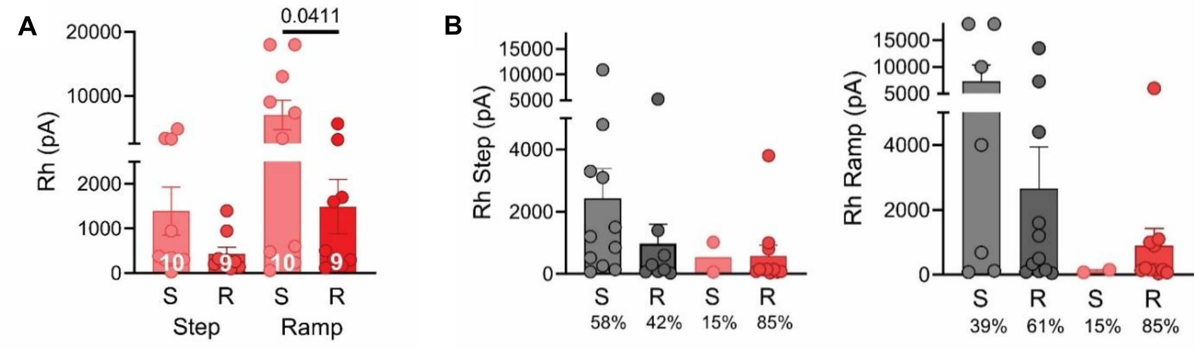

Supplemental Figure 2. **Rheobase in single vs repetitive firing neurons.** (**A**) Rh in non-responsive (S, single spiker) or responsive (R, repetitive spiker) cells to acute hIFN using step and ramp protocols. (**B**) Effect of prolonged exposure to hIFN on rheobase is shown for the percentage of S cells and R cells (shown at the bottom) with veh (gray bars) or IFN treatment (red bars) with step and ramp protocols. Data are presented as mean ± SEM. $p<0.05$ as determined by paired t-test in A, and unpaired t-test in the veh group in B.

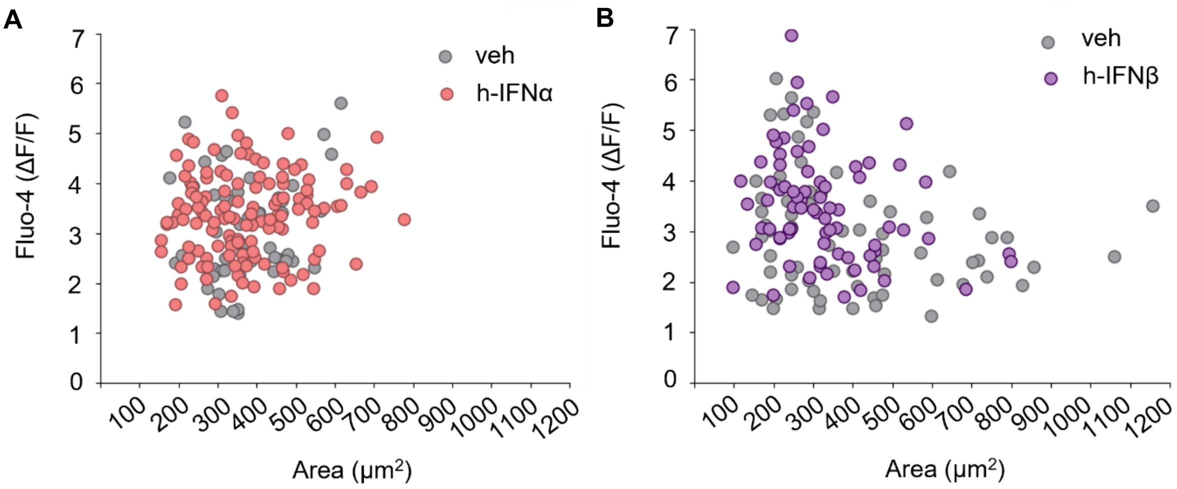

Supplemental Figure 3. Peak calcium response to capsaicin in hDRG neurons treated with hIFN-α or hIFN-β across different cell sizes. (**A**) Peak fold-change in calcium response to capsaicin of hDRG neurons treated with vehicle or hIFN-α plotted as a function of cell area. (**B**) Peak fold-change in calcium response to capsaicin of hDRG neurons treated with vehicle or hIFN-β plotted as a function of cell area.

Supplemental Table 1. Dorsal root ganglion donor demographic information

Assay	Donor	Age	Sex	Ethnicity	COD
RNAScope	1	29	F	White	Anoxia/Drug Overdose
	2	19	M	White	Anoxia/Drug Overdose
	3	30	M	Black	Anoxia/Asthma Attack
Treatments and ICCs	4	18	M	Black	Anoxia/Natural Causes
	5	52	M	White	CVA/Stroke
	6	22	M	White	Head Trauma/Blunt Injury/MVA
	7	37	F	Black	Head Trauma/GSW/Homicide
	8	49	M	White	CVA/Stroke
	9*	29	M	White	Head Trauma/Blunt Injury/MVA
	10	27	M	White	CVA/Stroke
	11	35	M	White	Anoxia/Drug Intoxication
	12	50	M	White/Hispanic	CVA/Stroke
Calcium Imaging	13	32	M	White	Head Trauma/MVA
	14	44	F	White	Anoxia/Cardiovascular
	15	33	M	White	Head Trauma
	16	46	M	White	Anoxia/Asphyxiation/Accident
	17*	25	M	Black	Anoxia secondary to MI
MEA	18*	28	M	White	Anoxia
	19*	49	M	White	Head Trauma/Blunt injury/MVA
	20*	18	M	Black	Head Trauma/GSW
Patch-clamp	21	45	M	White	Anoxia
	22	34	F	Black	Anoxia/Drug Overdose
	23	27	M	White	CVA/Stroke
	24	29	F	White	Head Trauma/GSW/Suicide
	25	11	M	Black	Anoxia
	26	20	M	White	Head Trauma/Blunt Injury/MVA
	27	19	M	White	Anoxia/Cardiovascular
	28	32	M	White	Anoxia/Drug Intoxication
	29	26	M	White/Hispanic	Anoxia/Drug Overdose
	30	26	M	White	Anoxia/Cardiovascular
	31	58	F	White	Anoxia
	32	50	M	White/Hispanic	Anoxia
	33	25	M	Black	Anoxia/Cardiovascular

* Indicates donors also used for patch-clamp experiments.

Supplemental Table 2. Passive and intrinsic membrane properties in hDRG neurons

	RMP (mV)	Rm (MW)	1st AP Th (mV)	1st AP Amp (mV)	1st Half-width (ms)	1st AHP Amp (mV)
**Acute**						
Pre-hIFN-α	-58.0 ± 2.2 (n=19)	170.8 ±36.7 (n=19)	-30.4 ± 2.8 (n=18)	86.3 ± 4.8 (n=19)	2.0 ± 0.25 (n=18)	-27.2 ± 1.31 (n=19)
Post-hIFN-α	NA	NA	-19.4 ** ± 4.4 (n=18)	75.2 * ± 5.0 (n=19)	3.1 ± 0.73 (n=18)	-22.6 * ± 2.6 (n=19)
**24-48 h Step**						
Veh	-54.7 ± 2.4 (n=27)	171.4 ± 26.8 (n=26)	-27.4 ± 4.7 (n=20)	75.3 ± 4.5 (n=20)	2.5 ± 0.4 (n=20)	-25.1 ± 1.8 (n=19)
hIFN-α	-54.5 ± 2.5 (n=23)	211.6 ± 33.7 (n=24)	-34.4 ± 2.5 (n=18)	87.7* ± 3.8 (n=18)	3.3 ± 0.5 (n=18)	-27.5 ± 1.8 (n=15)
**24-48 h Ramp**						
Veh			-26.9 ± 7.8 (n=15)	79.2 ± 3.8 (n=15)	3.4 ± 0.4 (n=15)	-24.5 ± 2.8 (n=15)
hIFN-α			-32.9 ± 2.9 (n=18)	83.6 ± 4.6 (n=18)	3.9 ± 0.5 (n=18)	-25.1 ± 1.5 (n=15)

Values correspond to the mean ± SEM. RMP = resting membrane potential; Rm =membrane resistance; 1st AP Th = threshold of the 1st action potential in a pulse; AHP = afterhyperpolarization. AP and AHP amplitudes were calculated from AP threshold. * p <0.05, ** p <0.01 as determined by paired t-test in acute experiments and unpaired t-test in 24-48 h hIFN-α exposure experiments.
